# Effect of place-based policy on regional economic growth: A quasi-natural experiment from China’s Old Revolutionary Development Program

**DOI:** 10.1371/journal.pone.0288901

**Published:** 2023-07-27

**Authors:** Dan Pan, Peiyao Zhou, Fanbin Kong

**Affiliations:** 1 School of Economics, Jiangxi University of Finance and Economics, Nanchang, Jiangxi, China; 2 China Academy of Public Finance and Public Policy, Central University of Finance and Economics, Beijing, China; 3 School of Economic Management, Nanjing Forestry University, Nanjing, Jiangsu, China; 4 School of Economic and Management, Zhejiang A&F University, Hangzhou, Zhejiang, China; Institute for Advanced Sustainability Studies, GERMANY

## Abstract

Triggering economic growth is a requirement to promote human welfare and realize sustainable development in many developing countries. However, place-based policies’ impact on economic growth is debatable, and its underlying mechanism is unknown. China’s Old Revolutionary Development Program (ORDP) is a large-scale and novel type of place-based policy targeted at undeveloped regions in China. We evaluate the effect of ORDP on economic growth by employing a time-varying difference-in-differences model and further explore the potential mechanisms and heterogeneity effects. VIIRS/DNB nightlight data is used to measure economic growth. We find that ORDP can significantly promote economic growth by 4.0% and the result is still robust after several tests. Mechanism analysis shows that ORDP can improve economic growth through government intervention, industrial structure optimization, and information infrastructure construction. Heterogeneity analysis indicates that the ORDP performs better on economic growth in central Chinese cities and high-economy cities. At the same time, our paper provides three practical suggestions for stimulating economic growth in ORDP, which can be enlightening for other developing countries.

## 1. Introduction

**Fig 1:**
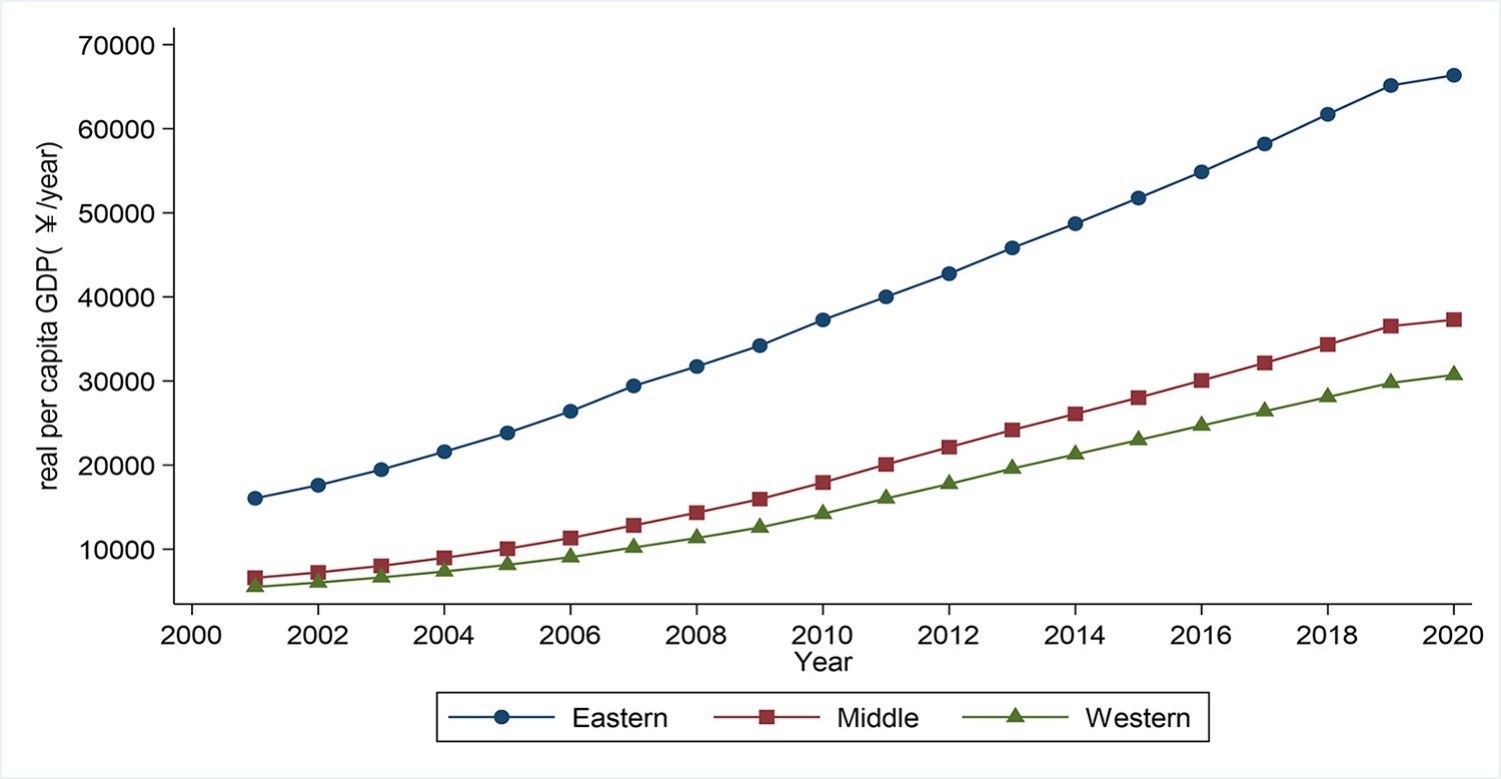
Differences in per capita GDP between three regions in China from 2001 to 2020. *Notes*: We classify cities in China into eastern, central, and western areas based on development level and geographic location [4]. The data comes from China Statistical Yearbook. Real per capita GDP is obtained by deflating per capita GDP using 2001 as the base year.

Regional disparity is a long-standing problem for both developed and developing nations [1]. China, the world’s biggest developing nation, has received widespread attention for unbalanced regional development [2]. Even though China has implemented a series of policies to address the regional disparity, such as the Central Rising Strategy and the Western Development Strategy, the economic divide between different geographical areas continues to widen. As shown in Fig 1, real per capita GDP differences among eastern, central, and western China are increasing over time. Specifically, eastern China’s real per capita GDP has grown much faster than central or western China’s, which could form a huge hidden barrier to China’s long-term economic growth [3]. Therefore, a better understanding of how to trigger economic growth in less developed areas and reduce regional disparity is a requirement to promote human welfare and realize sustainable development in China and other developing countries.

Place-based policies are spatially targeted and intended to promote economic growth in specific areas [5, 6]. Theoretically, place-based policies can stimulate economic growth in less developed areas and reduce regional disparity [7, 8]. However, in empirical studies, no consensus has been reached about place-based policies’ effect on the economy. Some studies prove that place-based policies help boost economic development [5, 9–11]. While another strand of literature holds the opposite view [4, 12–14]. Moreover, some scholars have found that place-based policies may benefit economic development in the short term, but it is unclear whether they will be effective in the long term [15, 16]. Hence, whether place-based policies can promote economic growth or not has not been confirmed and the underlying mechanisms of these two contradictory effects deserve attention. Therefore, we present the following research questions: Can place-based policies drive economic growth in targeted regions? Does this impact have heterogeneity in different regions? And what are the mechanisms behind it? Answering these issues is a prerequisite to designing place-based policies to spur economic development in developing countries including China.

To address the questions raised above, we make use of China’s Old Revolutionary Development Program policy (ORDP) as a form of place-based policy to capture the potential relationship between ORDP and economic growth. The ORDP seeks to encourage the growth and revitalization of the Old Revolutionary Base Areas by providing a range of supportive policies in fiscal, talent, and industry. It was implemented gradually, starting with 15 prefecture-level cities in 2012, followed by 10 cities in 2015 and 8 cities in 2016. Due to the limited availability of nightlight data which begins in 2013, we selected 18 prefecture-level cities that adopted ORDP in 2015 and 2016 as the experimental group. The control group consisted of 60 non-ORDP cities in the same provinces as the experimental group. Then, the Parallel Trend Assumption (PTA), which requires the control and experimental groups to share comparable economic development trends before the implementation of ORDP, was satisfied by doing this. We find that ORDP can increase economic growth by 4.0%. Further empirical results justify the robustness of our results. An impact mechanism analysis documents that ORDP improves economic performance mainly through government intervention, industrial structure optimization, and information infrastructure construction. Heterogeneity analysis implies that in terms of economic growth, ORDP performs better in central Chinese cities and high-economy cities.

Our paper makes the following four possible contributions. First, we supplement the burgeoning debate about whether place-based policies can improve economic growth. Although opinions on how place-based policies affect economic growth are divided, our research provides evidence that place-based policies do positively affect economic growth. As such, we offer valuable recommendations to optimize decision-making about place-based policies and China’s future regional development.

Second, we examine how China, the worldwide biggest emerging nation, is affected by place-based policies. According to Rafael et al. [17], place-based policies can alleviate market failures in developing economies such as lack of access to credit and financial services, ineffective resource distribution, and significant economic volatility. However, the institutional mechanism defects, corruption, and other drawbacks in developing economies may limit the effectiveness of these policies [18, 19]. For instance, in the case of China, Li et al. [31] identified the potential policy trap in place-based policy because of institutional mechanism defects. Therefore, the effect of place-based policies in developing economies is more complicated than that in developed economies. China is one of the most typical developing economies worldwide and ORDP in our paper is an example of place-based policies targeted at the backward areas in China. Therefore, we conduct a rigorous empirical test on how place-based policy can trigger economic growth in the context of backward areas in developing countries.

Third, we use VIIRS/DNB nightlight value to measure economic development, which can more accurately reflect real economic growth in China. China’s GDP, a commonly used measurement of economic development, has long been questioned by scholars [20]. In recent years, more and more scholars have used nighttime light data to denote economic development since this data is highly correlated with economic activities [21–24]. Using the nightlight value to represent economic development can avoid endogeneity among socio-economic statistical variables and eliminate human errors and distortions caused by political incentives [25]. However, in studies that examine place-based policies’ effect on economic growth, nightlight data is rarely utilized to characterize the economic level. Even when nightlight data is used, it is limited to DMSP/OLS nightlight data. Compared to DMSP/OLS stable nightlight data, VIIRS/DNB nightlight data has higher spatial and radiometric resolution and more precise temporal accuracy [26]. Therefore, we employ a more accurate nightlight value from VIIRS/DNB to measure economic growth.

Fourth, the DID method is utilized to rigorously test the relationship between ORDP and economic performance. Simple OLS estimation may bias the evaluation of policy effects [27], while DID method can reduce the estimation bias [15]. We test the net policy effect by using the ORDP as a semi-natural experiment. By doing this, we can lessen the endogeneity problem posed by omitted variables that affect both ORDP and economic growth, thus advancing methodological enlightenment for related research.

The arrangement of our paper is given below. Section 2 presents a literature review and an introduction to ORDP. Section 3 puts forward the theoretical structure and hypothesis. The figures and methodology are displayed in section 4. Section 5 discusses the empirical findings, including the average and dynamic treatment effect of ORDP on regional economic growth, a set of robustness tests, mechanism analysis, and heterogeneity analysis. Section 6 concludes and proposes corresponding policy suggestions.

## 2. Literature review and institutional background

### 2.1 Literature review

Place-based policies have become increasingly popular over the past several decades [5]. Many governments worldwide have implemented various place-based policies to promote coordinated regional development, such as the UK Regional Selective Assistance Policy [27], the European Union Structural and Cohesion Funds [28], the America Tennessee Valley Authority Policy [29], and the German Innovative Regional Growth Cores Program [30].

In the context of China’s Reform and Opening-up, the central government launched a range of place-based policies to promote economic growth. Many studies argue that place-based policy can improve economic performance. For example, Ren et al. [9] observe that there is a 25.70% increase in regional economic growth after the Northeast China Revitalization Strategy implementation. Wang et al. [10] find the Chinese National Economic and Technological Development Zones can also promote urban economic development. Jia et al. [11] employ a spatially discontinuous design and conclude that China’s Great Western Development Program contributes 1.6% to the annual GDP growth rate of target regions. The Special Zone Program is also found to be successful in accelerating a series of social-economic indicators such as employment, capital investment, output, and so on, thus improving the economic performance in the designated areas [5]. However, some scholars hold the opposite view. For instance, the National Poor Counties program has been proven to fail to stimulate local economic growth and the local capture should be part of the reason for the failure [12]. Yang et al. [4] argue that Regional Development Plans have not improved economic performance in the western area and even significantly hinder the eastern and central areas’ economic growth. Zhang et al. [13] demonstrate that the Western Development Strategy has not significantly promoted economic growth in the western region. Yang et al. [14] also suggest that Northeast China Revitalization Policy has not positively contributed to the long-term economic development of Northeast China overall. Another group of scholars combines these two views and argues that place-based policies in China are effective in their initial stages, but fail to sustain over time [15, 25, 31].

Combining the aforementioned literature, we can see that there is no consensus regarding how place-based policies affect China’s economic growth currently. This paper attempts to complement previous research by making use of ORDP as a counterfactual test, to confirm place-based policies’ economic impact in target regions.

### 2.2 Institutional background: ORDP in China

The term "Old Revolutionary Base Areas" (ORBAs) refers to the areas established by the Chinese Communist Party during the Anti-Japanese War as well as the Agrarian Revolutionary War. As a result of the great sacrifices in the victory of the Chinese revolution and important contributions made to socialist construction, ORBAs experienced significant economic decline. In addition, the poor geographical conditions, inadequate transportation infrastructure, and limited access to information have significantly hindered the development of ORBAs and made it harder for residents to catch up with other regions [32].

As Chinese President Xi Jinping stressed, "We cannot forget history, we cannot forget the martyrs and heroes who fought for the birth of the new China, and we cannot forget the people of the old revolutionary areas who made significant contributions to the revolution." To let people in ORBAs have a happier and better life, relevant departments have drafted and promoted a range of ORBAs’ supportive policies, and finally formed a "1258" policy system. This system includes 1 general guiding opinion, 2 regional policy opinions, 5 key ORBAs revitalization and development plans, and 8 area regional development and poverty alleviation plans [32]. Of these policies, "5" refers to the Old Revolutionary Development Policy strategies (ORDP) on five inter-provincial ORBAs, namely Shaanxi-Gansu-Ningxia ORBA, Gansu-Fujian-Guangdong ORBA, Dabie Mountain ORBA, Left and Right Rivers ORBA, and Sichuan-Shaanxi ORBA. In the year of 2021, the State Council released its "Opinions on Supporting the Revitalization and Development of ORBAs in the New Era" to continuously promote the construction of these five ORBAs. Overall, the ORDP cities covered 7.18% of China’s area (690 thousand square kilometers), 11.15% of its population (150 million), and 6.25% of its gross domestic product in 2012.

Aiming at the revitalization and development of ORBAs, ORDP includes a series of fiscal, land, talent, and industrial policies, which have great impacts on economic growth. For example, ORDP proposes that central and local financial investments should be given priority to ORBAs, which can promote economic growth in target areas [33]. Moreover, ORDP emphasizes the development of some local special industries, which are important for regional economic growth. For instance, ORDP in Dabie Mountain points out that in the cities of Xinyang and Zhumadian, a national quality inspection center for non-metals and spices should be supported to build [10].

## 3. Theoretical framework

### 3.1 The impact of ORDP on regional economic growth

As we mentioned in the introduction section, the economic performance of place-based policies is controversial as it can either facilitate or hinder economic growth. However, based on the specific measures implemented in ORDP areas, we put forward Hypothesis 1 that ORDP can facilitate the economic growth of the target regions. This is because as a national initiative to promote the development of ORBAs, ORDP is accompanied by a series of preferential policies such as increasing financial investment, supporting industrial development, and enhancing human capital through the inflow of talent. As a result, it can promote economic growth. For example, increased financial investment can initially guarantee the economic development of undeveloped areas [25], talent policy makes up for the shortage of professional and technical talents in backward regions [34], and industrial development promotes regional industrial structure optimization [10], which in turn promotes economic growth. Therefore, we reckon that ORBAs can benefit from these policy tilts directed by ORDP and then achieve economic growth.

We also propose Hypothesis 2 that the effect of ORDP on the economy will take a specific period of time to emerge and may fail to sustain in the long run. There are two reasons for this. First, the policies implemented in the ORDP require a certain amount of time for local governments and companies to respond, so it will take some time for the effects of ORDP to become apparent. Second, ORDP achieves its positive policy effects mainly through large-scale physical investments, such as infrastructure construction and physical investments, rather than through technological inputs or human capital investments. According to Solow Growth Model, this development pattern has a short-term impact on economic development but may gradually diminish or even disappear over time.

### 3.2 The mechanism of ORDP on regional economic growth

According to the above analysis, we hypothesize that ORDP can effectively promote economic growth, but clarifying the mechanism behind it is equally enlightening for the future development path of the ORDP. Combining the prior literature, we propose Hypothesis 3 that ORDP facilitates economic growth mainly through government intervention, industrial structure upgrading as well as information infrastructure construction. The reasons are given below.

First, ORDP will increase local government intervention, leading to growth in financial input and the improvement of resource allocation efficiency. This, in turn, promotes economic growth. ORDP issues a series of government intervention policies with abundant financial investment to the ORBAs. It is reported that in the context of the "Chinese 13th Five-Year Plan", 1/8 of the central funds are used in ORBAs, with an investment scale of more than 300 billion yuan. This massive financial investment is beneficial to economic growth. For one reason, financial support including transfer payments, tax benefits, and fee reductions enable local governments to improve the ORBAs’ income growth [35]. For another reason, increasing financial input helps ameliorate market failure and optimize resource allocation [36]. Besides, for the backward regions, financial investment can provide an initial financial guarantee for their future development and push their economic growth [25].

Second, ORDP promotes the optimization of industrial structure, which in turn improves labor productivity and leads to overall economic development. There are two reasons for this. Firstly, ORDP encourages the development of advantageous industries based on local resource endowments. For example, the revitalization plan for Dabie Mountain Revolutionary Area clearly states to support the construction of a national non-metal and spices quality inspection center. Developing advantageous industries can improve resource allocation and thus promote the productivity of enterprises, which ultimately contributes to economic development [8]. Secondly, ORDP can benefit from agglomeration economies. ORDP cities are designed as a group, which indicates that firms in these regions can share intermediate inputs, labor pools, and other production factors to accelerate the upgrading of industrial structures and finally stimulate economic growth [10].

Third, ORDP can boost information infrastructure development on a large scale, which can introduce new technologies to drive innovation and reduce transaction costs as well as information asymmetry in economic activities, thereby facilitating economic growth. Modern information technologies, such as modern agricultural information technology, 5G mobile communication networks, e-commerce, big data, and cloud computing, enable manufacturers to better allocate production factors between different industrial sectors. For example, in the agriculture sector, the deep integration of modern information technology with agriculture can generate a new agricultural production model and promote agricultural development. Also, rural e-commerce, a new engine for rural economic development, is accompanied by the improvement of information infrastructure construction [37]. Furthermore, information infrastructure serves as a lubricant in socio-economic activities, reducing the transaction costs and information asymmetry of economic activities and enhancing the efficiency of economic activities [14, 38].

## 4. Data and method

### 4.1 Data and variables

Before conducting empirical test, the Nighttime Satellite Light Data, ORDP data, and other variables influencing economic growth are manually collected. The final sample is made up of data from 78 cities spread throughout three old revolutionary areas, namely Sichuan-Shaanxi ORBA, Dabie Mountain ORBA, and Left and Right River ORBA, from 2013 to 2019.

We use a sample of 78 prefecture-level cities located in three ORBAs because they implemented ORDP after 2013. In contrast, the other two ORBAs implemented ORDP before 2013. Specifically, Sichuan-Shaanxi implemented ORDP in August 2016, Dabie Mountain ORDP was implemented in July 2015, and the Left and Right River ORDP was executed in March 2015. The reasons why we select the period from 2013 to 2019 as our sample are as follows. First, the VIIRS/DNB Nighttime Satellite Light Data is only available since 2013, so we perform regression using the sample beginning in 2013 [14, 26]. Second, most data after 2019 is inaccessible due to data availability. For example, the nightlight value is only available before 2019.

#### 4.1.1 The Nighttime Satellite Light Data

The VIIRS/DNB Nighttime Satellite Light Data is used to measure economic growth. These data come from the China Research Data Service Platform (CNRDS). To decrease heteroscedasticity, nightlight values are converted to a natural logarithmic form.

#### 4.1.2 ORDP data

We select ORDP as the independent variable. If ORDP is implemented in city *i* of year *t*, then we define ORDP for the city in year *t* and subsequent years as 1, and 0 otherwise.

#### 4.1.3 Control variables

Based on existing studies, our research chooses a set of control variables to control possible factors that may impact economic growth. Consequently, five control variables are selected: consumption, population density, human capital, financial development, and foreign investment.

1. Consumption. As defined by the proportion of total social consumption to GDP. Consumption is one of the driving forces of economic growth [9]. One reason is that residents with high consumption levels have sufficient purchasing power and generate a consumption-driven effect that will ultimately boost economic development [39]. Another reason is that the improvement of residents’ consumption ability can lead to an overall increase in regional consumption level, which can attract high-quality talent. This talent is one of the key drivers of economic development [39, 40].
2. Population density. We convert the population per square kilometer into logarithmic form to represent population density. The relation between population density and economic growth is not linear but inverted U-shaped [41]. In other words, when the population density is lower than a certain threshold, it can supply the necessary human resources for economic growth. However, once the population density exceeds the environment’s capacity to create food and energy required for human livelihood, the pace of economic development will slow down [10].
3. Human capital. This variable is defined as the total number of college students. Skilled labor has been regarded as an endogenous force for economic growth [42]. This is because human capital can increase productivity by developing workers’ skills and encouraging technology spillovers [43].
4. Financial development. As determined by the proportion of the year-end loanable fund to GDP. Financial development can positively facilitate economic growth [44]. According to Marco [45], a well-performed financial system contributes to the reduction of information asymmetry, mobilization of savings, allocation of funds, strengthening of corporate governance, and risk diversification. In this way, resources can be allocated where they can yield optimized results and promote economic growth.
5. Foreign investment. We measure this variable using the natural logarithm of foreign direct investment (FDI). It is debatable how FDI affects regional economic expansion. For one thing, FDI can encourage the growth of local businesses by introducing cutting-edge technologies and innovative management methods, thereby having a good effect on regional economic expansion [10]. For another, FDI might hinder economic development. The massive inflow of FDI has contributed to the rapid development of multinational corporations (MNCs). To maintain their competitive advantage, MNCs will suppress local enterprises, leading to social efficiency loss and impeding local economic development [9].

The China City Statistical Yearbook and the China Provincial Statistical Yearbook are the sources of all these indicators. The average growth rate is used to supplement the missing data. Consequently, the panel data of 546 samples of 78 cities from 2013 to 2019 are finally derived.

Table 1 displays the descriptive statistics and definitions for all these variables.

**Table 1:** Statistics and explanations for the sample variables.

Variables	Definition	N	Mean	S.D.
Nightlight	Proxy variable for economic growth, measured by Ln (average nightlight digital number per year)	546	0.426	0.468
ORDP	= 1, if a city has implemented ORDP; = 0 otherwise	546	0.150	0.357
Consumption	The proportion of total social consumption to GDP (%)	546	0.396	0.093
Population density	Ln (population per square kilometer) (hundred people/km^2^)	546	0.050	0.029
Human capital	The number of college students (billion people)	546	2.475	2.161
Financial development	The ratio of the year-end loanable fund to GDP (%)	546	1.165	0.432
Foreign investment	Ln (real FDI based on 2013) (CNY)	546	11.652	1.812

### 4.2 Model: Time-varying DID method

Endogenous problem is a great challenge for policy effect identification, and DID model can eliminate the endogenous problem by comparing the group affected by the policy to that unaffected over time [46]. Therefore, we apply a DID method to assess the possible relation between ORDP and economic growth. Considering the different implementation times of ORDP, we employ the time-varying DID method, which enables us to control for time-variant, city-level omitted characteristics by including city-specific dummy variables [47]. We set the fundamental time-varying DID model shown as follows:

$${nightlight}_{it}=\alpha_{0}+\alpha_{1}{ORDP}_{it}+{\gamma X_{it}+\delta }_{i}+\theta_{t}+\varepsilon_{it}$$ (1)

*nightlight*_*it*_ is a proxy variable of economic growth of city *i* in year *t*. If city *i* implemented ORDP in year *t*, *ORDP*_*it*_ equals 1, and 0 otherwise. Therefore, *α*_1_ assesses the net impact of ORDP on economic growth. If *α*_1_ is positive and significant, it suggests that ORDP increases economic growth. *X*_*it*_ represents the control variables shown in Table 1. *δ*_*i*_ reflects the city-fixed effect and *θ*_*t*_ is the year-fixed effect. *α*_0_ is the intercept, *ε*_*it*_ is the error term.

After estimating the average treatment effect of ORDP on economic growth, we examine the dynamic treatment effect and test the common trend assumption. Specifically, we modify the model based on Louis et al. [48] to conduct this process, which can be denoted as:

$${nightlight}_{it}=\alpha_{0}+\sum_{k\geq -2}^{4}\alpha_{k}{ORDP}_{it}^{k}+\gamma X_{it}+\rho {treat}_{i}+\sigma {prepost}_{t}+\varepsilon_{it}$$ (2)

If city *i* is one of the ORDP cities, then *treat*_*i*_ equals 1, and otherwise 0. Similarly, *prepost*_*t*_ equals 1 if the year is after the implementation year of ORDP and otherwise 0. The dummy variables ${ORDP}_{it}^{k}$ is the interaction term between *treat*_*i*_ and *prepost*_*t*_ and is corresponding to 2013, 2014, 2015, 2016, 2017, 2018, and 2019. Specifically, *k* = 0 denotes the year when the city implemented ORDP, which is treated as the base period. *α*_*k*_ indicates the net policy impact in year k. The rest variables’ meanings are the same as those in Formula (1).

## 5. Empirical results

We first perform the common trend test and then demonstrate the ORDP’s average treatment effect on economic growth based on regression on Eq (1), followed by a dynamic treatment effect of ORDP using Eq (2). We also conduct seven robustness tests to verify the robustness of base results. Then, three possible mechanisms of ORDP on economic growth are investigated. Finally, we evaluate the heterogeneous effects of ORDP on economic growth.

### 5.1 Common trend test

Before using the DID approach to estimate the ORDP’s effect on economic growth, we should pre-test whether the economic growth in ORDP and non-ORDP cities shows a common trend prior to ORDP being implemented. Therefore, in this part, we examine the pre-existing trend based on Eq (2).

**Fig 2:**
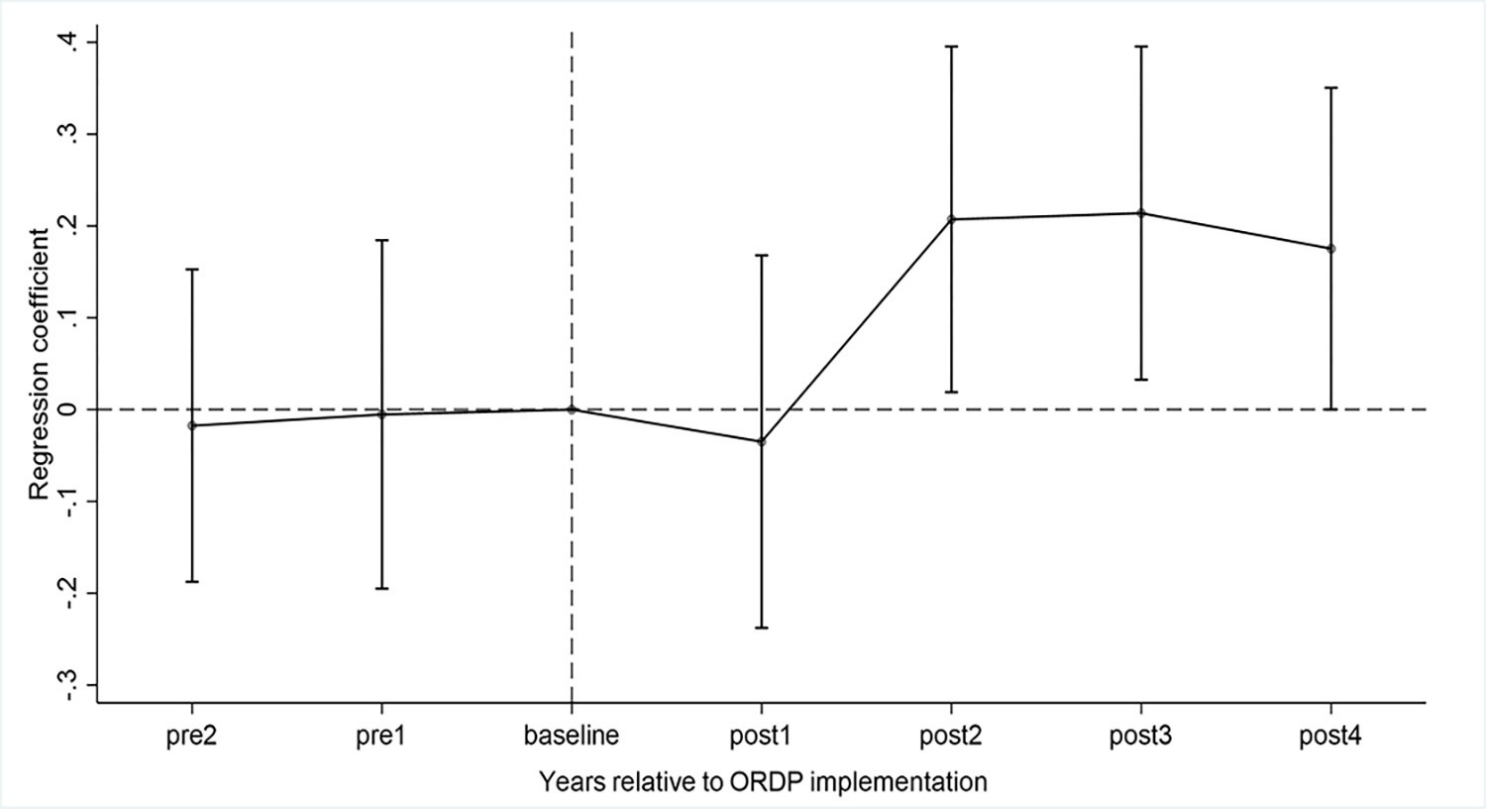
Common trend test.

As shown in Fig 2, we can find that before the city implements the ORDP, the estimated coefficients of ORDP are all insignificantly different from zero, meeting the common trend assumption.

### 5.2 The average treatment effect of ORDP on economic growth

The average treatment effect of ORDP on economic growth is shown in Table 1. It can be found that ORDP significantly facilitates economic growth by 4.0%. Therefore, we conclude that ORDP can increase economic development in ORDP areas, which verifies our Hypothesis 1.

### 5.3 The dynamic treatment effect of ORDP on economic growth

From a static perspective, the benchmark regression shows that ORDP significantly and positively affects economic growth, however, if this impact will be long-lasting is unclear. Thus, according to Beck et al. [47], we identify dynamic treatment effects and Table 2 shows the results.

**Table 2:** The average and dynamic treatment effect of ORDP on economic growth.

	Average treatment effect	Dynamic treatment effect
ORDP	0.040**(0.019)	
Consumption	-0.222(0.164)	0.375*(0.199)
Population density	3.189(3.285)	3.933***(0.640)
Human capital	0.020*(0.010)	0.121***(0.012)
Financial development	0.052(0.038)	0.033(0.052)
Foreign investment	0.013**(0.006)	-0.011(0.010)
*ORDP*_−2_		-0.018(0.103)
*ORDP*_−1_		-0.005(0.115)
*ORDP*_1_		-0.035(0.123)
*ORDP*_2_		0.207*(0.114)
*ORDP*_3_		0.214*(0.110)
*ORDP*_4_		0.175*(0.107)
*treat*_*i*_		-0.077(0.081)
*prepost*_*t*_		0.167***(0.033)
City fixed effect	Yes	No
Year fixed effect	Yes	No
R^2^	0.962	0.513
Observations	546	546

*Notes*: (1) What in parentheses are the robust standard errors clustered by cities; (2) ***, **, and * denote statistical significance of 1%, 5%, and 10%, respectively.

It is necessary to note that the sample for the 3^rd^ year before ORDP implementation is only 15 and we have grouped them into the 2^nd^ year before ORDP implementation. In addition, the ORDP implementation year is taken as the baseline year. According to the results, we can conclude that the policy effect of ORDP takes time to emerge and is not long-lasting, which validates our Hypothesis 2. Specifically, the coefficient of *ORDP*_1_ is not significant, suggesting that in the first year after ORDP implementation, there is no impact on economic progress. The ORDP’s effect on economic development, however, appears to increase in the 2^nd^, 3^rd^, and 4^th^ years following its implementation, in that the coefficients of *ORDP*_2_, *ORDP*_3_, and *ORDP*_4_ are significant. However, the positive trend of ORDP’s impact on economic growth is not sustainable, as ORDP_4_′s coefficient (0.175) is smaller than that of ORDP_3_ (0.214).

### 5.4 Robustness tests

We also conduct several robustness tests. These specifically include the following seven: a placebo test, replacing the dependent variable with real per capita GDP, excluding the impact of TPA policy, excluding the provincial and sub-provincial cities, using the propensity score matching and the difference-in-differences model (PSM-DID), changing the sample period, and excluding the effect of outliers.

#### 5.4.1 Placebo test

We further carried out two placebo tests to see if our baseline regression results excluded random probability. One placebo examination is conducted counterfactual time. Specifically, we advance the ORDP implementation year by 1 year to construct a false ORDP implementation time. If the coefficient of false ORDP is insignificant, then the economic growth is indeed caused by ORDP. Therefore, we are able to rule out any potential influence from other factors prior to ORDP. The insignificant coefficient of false ORDP in Table 3 suggests that the ORDP does lead to an increase in economic growth.

**Table 3:** Placebo test results.

	The Year of ORDP implementation	Advance ORDP by 1 year
ORDP	0.040**(0.019)	
Advance ORDP by 1 year		0.010(0.028)
Control variables	Yes	Yes
City fix effect	Yes	Yes
Year fix effect	Yes	Yes
R^2^	0.962	0.962
Observations	546	546

*Notes*: The same as Table 2.

We conduct counterfactual samples as the second placebo test. Specifically, following Raj et al. [49], we choose 18 cities at random to represent the pseudo-ORDP group and the remaining as the non-ORDP group, then run regression based on the counterfactual ORDP samples. To improve reliability, we repeat the procedure 500 times and record the estimated parameters. According to Jiang et al. [25], the parameter $\hat{\beta_{1}}$ is estimated based on Eq (3).


$$\hat{\beta_{1}}=\beta_{1}+\gamma \frac{cov({ORDP}_{it},\mu_{it}|control)}{var({ORDP}_{it}|control)}$$ (3)

Where "control" denotes the control variables. If the estimate of $\hat{\beta_{1}}$ is unbiased, then *γ* needs to be 0. Since it is difficult to determine whether *γ* is 0 and to directly test whether the estimated coefficient of $\hat{\beta_{1}}$ is affected by the unobservable random perturbation term, this paper randomly assigns ORDP dummy variables. If $\hat{\beta_{1}}$ still equals 0, then we can deduce that *γ* is 0.

**Fig 3:**
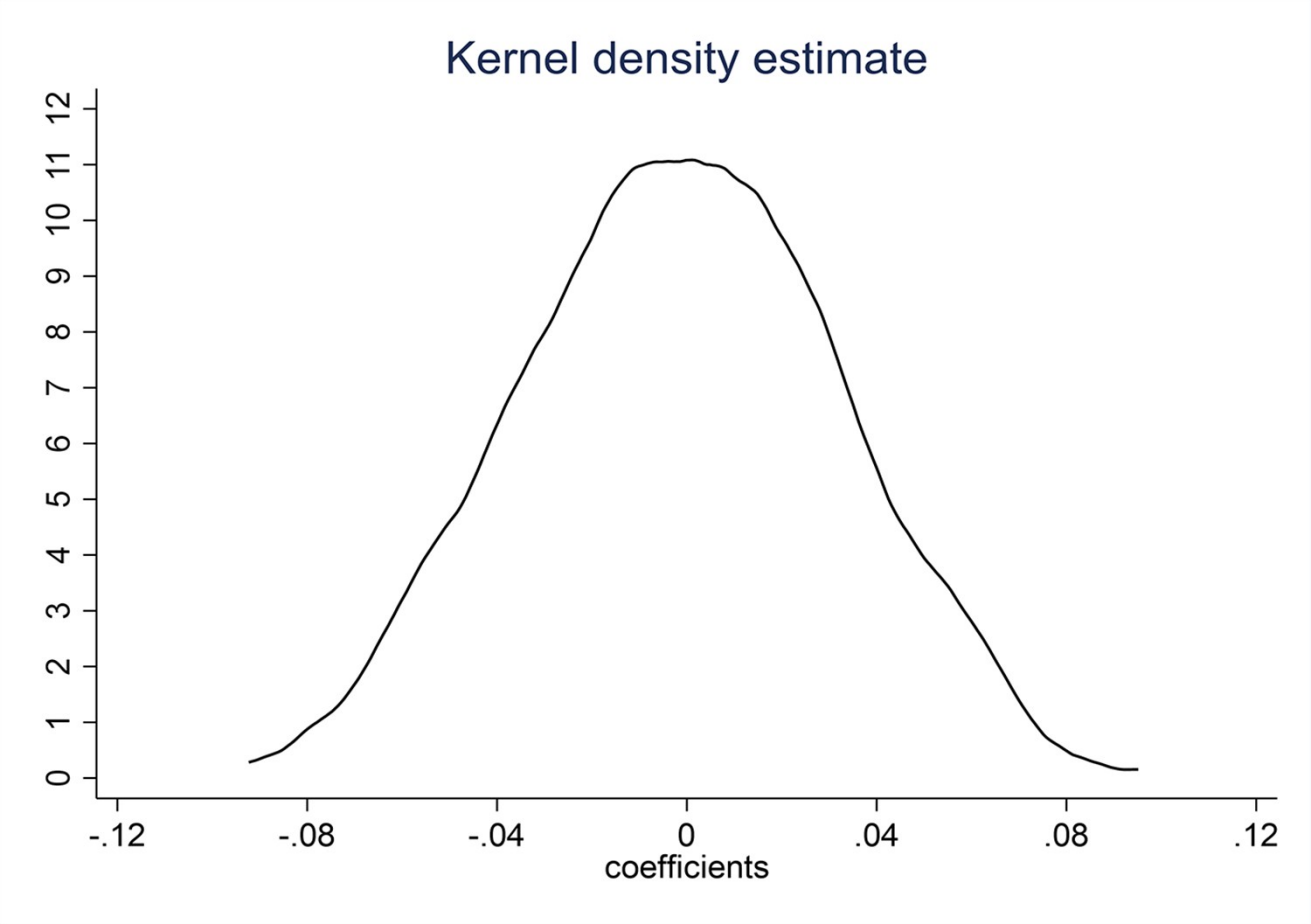
The regression coefficients’ distribution using pseudo-samples.

Fig 3 illustrates the coefficient distribution for the pseudo-ORDP cities. It can be found that the estimated coefficients are mostly concentrated near zero, which allows us to infer that *γ* is 0. Therefore, the ORBAs’ impact on economic development is unaffected by the random assignment sample, proving the ORDP policy’s actual positive effect on economic development.

#### 5.4.2 Replacing the dependent variable

We replace the VIIRS/DNB nightlight value with real per capita GDP to evaluate economic growth, since it is a common measurement of economic growth by scholars. It should be noted that real per capita GDP is converted into natural logarithmic form. After the replacement of the dependent variable, the coefficient of ORDP, which is shown in Table 4, is still significantly positive, further demonstrating ORDP’s driving effect on economic growth.

**Table 4:** The results of several robustness tests.

	Baseline result	Using real per capita GDP as the dependent variable	Exclude the effect of the TPA policy	Exclude provincial and sub-provincial cities	Using PSM-DID method
ORDP	0.040**	0.291***	0.053***	0.035*	0.057**
	0.019	0.098	0.020	0.020	0.023
TPA			-0.043*		
			0.010		
Control variables	Yes	Yes	Yes	Yes	Yes
City fix effect	Yes	Yes	Yes	Yes	Yes
Year fix effect	Yes	Yes	Yes	Yes	Yes
R^2^	0.962	0.242	0.962	0.942	0.947
Observations	546	546	546	504	390

*Notes*: The same as Table 2.

#### 5.4.3 Excluding the impact of the TPA policy

In 2014, a place-based policy in China, the Targeted Poverty Alleviation (TPA) Policy was also proposed in addition to the ORDP. Not only was the TPA policy implemented at roughly the same time as ORDP, but many of the TPA cities also matched the ORDP cities. To improve the accuracy of our baseline result, we need to exclude the impact of the TPA policy. Specifically, we construct the following model by adding TPA policy as a dummy variable to our benchmark DID model:

$${nightlight}_{it}=\alpha_{0}+\alpha_{1}{ORDP}_{it}+\alpha_{2}{TPA}_{it}+{\gamma X_{it}+\delta }_{i}+\theta_{t}+\varepsilon_{it}$$ (4)

*TPA*_*it*_ equals 1 if city *i* implemented TPA policy in year *t*, and 0 otherwise. Other variables are the same as they are in the benchmark model.

Table 4 displays the results, where the symbol and significance of *ORDP*_*it*_ are in line with the outcome of our baseline model, indicating the accuracy of our estimates.

#### 5.4.4 Excluding the provincial and sub-provincial cities

The potential factors affecting economic development may vary depending on the city’s administrative hierarchy, thus leading to a biased estimation of the net policy effect [50]. For instance, Gao et al. [51] demonstrate that cities with higher hierarchical levels, such as provincial and sub-provincial cities, have access to more preferential policies and can influence the distribution of resources to some extent. Although most of our study samples are ordinary cities, they still include some provincial and sub-provincial cities. After removing six provincial and sub-provincial cities, we performed regression following Jiang et al. [25]. We can discover from Table 4 that the ORDP’s coefficient slightly lowers but remains statistically significantly positive, backing up our basic findings.

#### 5.4.5 Using the PSM method

**Fig 4:**
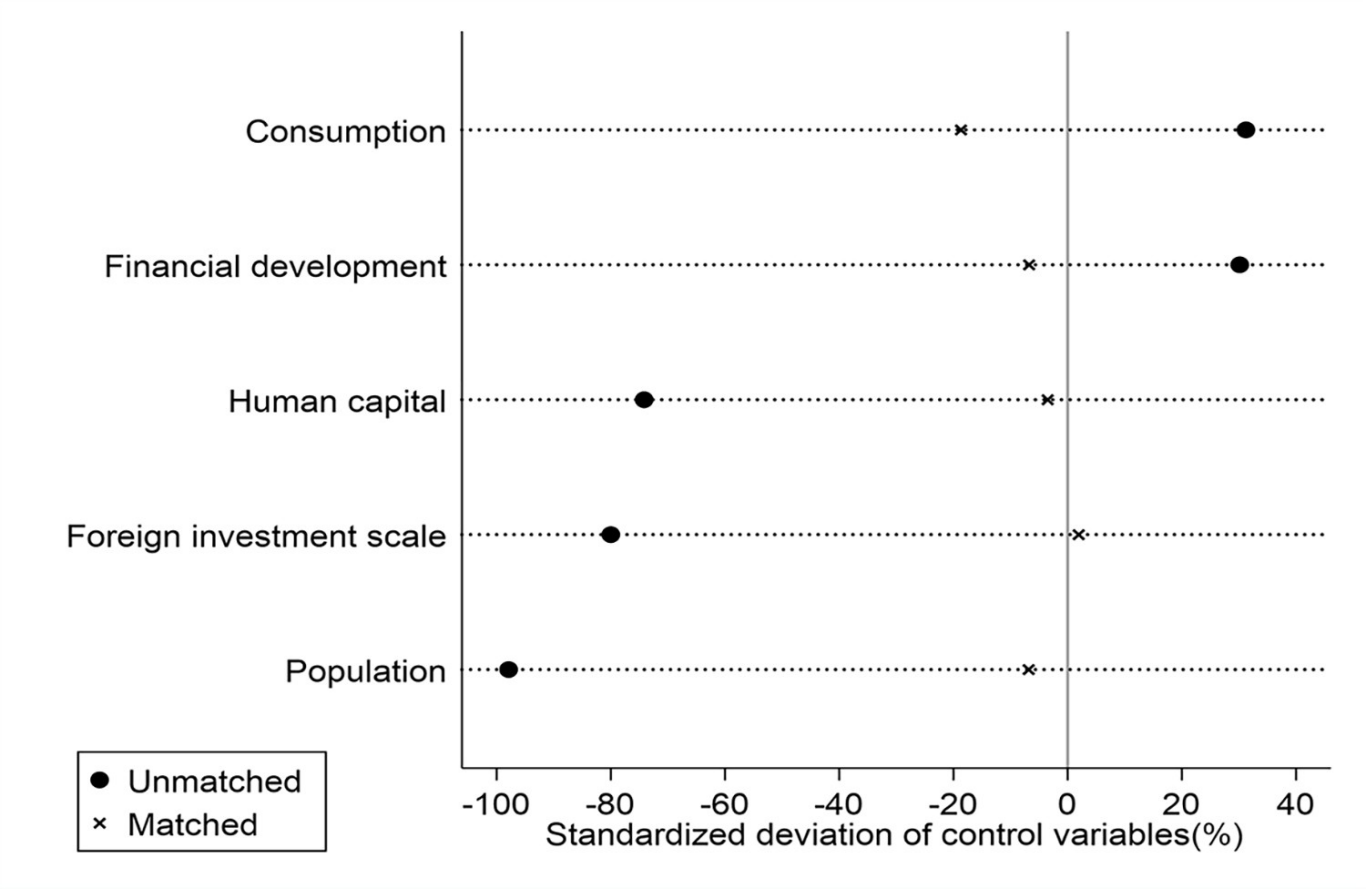
The standardized deviation of control variables before and after PSM.

The combination of PSM and DID methods is first proposed by James et al. [52] to identify control groups. In accordance with Yang et al. [50], the following steps are taken. First, the propensity score is computed based on the logit model for each sample. Second, the ORDP city is matched to the non-ORDP city by using the 1 nearest neighbor matching method. Third, carrying out the common value test as well as the matching balance test and the results are shown in Fig 4. We can see that all control variables’ standardized deviations are less than 20% after matching than they were before, showing that the matching meets the balance test. Finally, after passing the common value and matching balance test, we then re-estimate the net policy effects using the matched samples. It can be found from Table 4 that ORDP still significantly boosts economic growth by 5.7%, which is in line with the baseline findings and adds to the validity of our conclusions.

#### 5.4.6 Changing the sample period

In order to eliminate the possible influence of the COVID-19 pandemic in 2019, following Pan et al. [46], we shorten the time interval from 2013–2019 to 2014–2018, which is one year before and after the policy than the original sample. Table 5‘s findings reveal that the coefficients on ORDP remain significantly positive, proving that the period setting has no impact on the policy effect.

**Table 5:** Results of changing the sample period.

	2013–2019	2014–2018
ORDP	0.040**(0.019)	0.047**(0.021)
Control variables	Yes	Yes
City fix effect	Yes	Yes
Year fix effect	Yes	Yes
R^2^	0.962	0.971
Observations	546	390

*Notes*: The same as Table 2.

#### 5.4.7 Excluding outliers

Extreme values are another potential issue that could jeopardize the benchmark findings. The histograms of data reveal that the outliers are generally distributed on the right side of the data set. Therefore, following Pan et al. [53], we winsorize the up 1%, 3%, and 5% of the samples and repeat the benchmark regression. The outcome in Table 6 demonstrates that the coefficients of ORDP are comparable to the baseline findings. Thus, providing another verification of our conclusions’ robustness.

**Table 6:** Results of winsorizing extreme values.

	Winsorizing extreme value (1%)	Winsorizing extreme value (3%)	Winsorizing extreme value (5%)
ORDP	0.043**(0.019)	0.047**(0.019)	0.048**(0.019)
Control variables	Yes	Yes	Yes
City fix effect	Yes	Yes	Yes
Year fix effect	Yes	Yes	Yes
R^2^	0.960	0.956	0.945
Observations	546	546	546

*Notes*: The same as Table 2.

### 5.5 Mechanism analysis

The above results have proven that ORDP can boost economic growth. However, what mechanism behind ORDP remains to be answered. As we stated in the theoretical framework, economic performance can be improved by enhancing government intervention, optimizing industrial structure, and strengthening information infrastructure investment.

To check these three possible mechanisms, in accordance with Baron et al. [54], the following method is developed:

$${nightlight}_{it}=\alpha_{0}+\alpha_{1}{ORDP}_{it}+{\gamma X_{it}+\delta }_{i}+\theta_{t}+\varepsilon_{it}$$ (5)

$$M_{it}=\beta_{0}+\beta_{1}{ORDP}_{it}+{\gamma X_{it}+\delta }_{i}+\theta_{t}+\varepsilon_{it}$$ (6)

$${nightlight}_{it}=\tau_{0}+\tau_{1}{ORDP}_{it}+\tau_{2}M_{it}+{\gamma X_{it}+\delta }_{i}+\theta_{t}+\varepsilon_{it}$$ (7)

Where *M*_*it*_ represents three mediation variables: government intervention, industrial structure optimization, and information infrastructure construction. We use the ratio of government expending to GDP to stand for government intervention. The greater the value, the more forceful the government intervention is [33]. Regarding industrial structure optimization, following Zheng et al. [55], we use the industrial structure upgrading index to denote it, which is created by the ratios of the primary, secondary, and tertiary industry to GDP multiplied by 1, 2, and 3, respectively. The natural logarithm of the number of internet access users denotes the level of information infrastructure [56]. *α*_1_ is the total effect of ORDP on economic growth; *τ*_1_ represents the direct influence of ORDP on economic development; *β*_1_×*τ*_2_ indicates the indirect effect of ORDP on economic growth, *γ*, *δ*_*i*_, *θ*_*t*_, *ε*_*it*_, and *X*_*it*_ are the same as the benchmark model.

We judge whether there is a mediation effect according to the following three steps [46]. First, assess the overall effect *α*_1_, and if it is significant, move on to the next step. Second, test *β*_1_ and *τ*_2_. In case both coefficients are significant, test *τ*_1_. A significant *τ*_1_ indicates a partial mediation effect, while a non-significant *τ*_1_ indicates a full mediation effect. Third, in the case that at least one of *β*_1_ and *τ*_2_ is insignificant, the Sobel test is executed. If three conditions are satisfied, i.e., the coefficient of the Sobel coefficient is significant, *τ*_1_ is significant, and *β*_1_×*τ*_2_ has the same sign as *τ*_1_, then there is a partial mediation effect, and a full mediation effect exists if only the Sobel coefficient is significant and *τ*_1_ is insignificant, otherwise, there is no mediation effect.

Table 7 displays the findings of the mechanism analysis. The overall impact is seen in Column 1. Three mechanisms are arranged in columns 2 to 7 sequentially.

**Table 7:** Three mechanisms of ORDP on economic growth.

	Total Effect	Government Intervention		Industrial Structure Optimization		Information Infrastructure Construction	
	Night-light	Government Intervention	Night-light	Industrial Structure Optimization	Night-light	Information Infrastructure Construction	Night-light
ORDP	0.040**	0.045***	0.032	0.022	0.037*	0.044	0.037**
	(0.019)	(0.009)	(0.020)	(0.014)	(0.019)	(0.037)	(0.019)
Government Intervention			0.171 (0.165)				
Industrial Structure Optimization					0.143* (0.076)		
Information Infrastructure Construction							0.066** (0.029)
Control	Yes	Yes	Yes	Yes	Yes	Yes	Yes
City F.E.	Yes	Yes	Yes	Yes	Yes	Yes	Yes
Year F.E.	Yes	Yes	Yes	Yes	Yes	Yes	Yes
Sobel test	-	0.072***(0.017)		0.027**(0.014)		0.060***(0.017)	
R^2^	0.962	0.808	0.962	0.745	0.963	0.931	0.963
Observations	546	546	546	546	546	546	546

*Notes*: The same as Table 2.

First, two columns entitled "Government Intervention" show our first mechanism. It can be seen that the effect of ORDP on government intervention *β*_1_ (0.045) is significant and positive, which is consistent with our expectations. However, despite being positive, the coefficient of government intervention *τ*_2_ (0.171) is not significant. Therefore, a Sobel test is conducted and the result (0.073) is significantly favorable, proving the existence of a government intervention mediating mechanism.

Second, the following two columns labeled with "Industrial Structure Optimization" are the second mechanism. Industrial structure’s coefficient *τ*_2_ (0.143) is positive and significant, while ORDP’s influence on industry structure (*β*_1_) does not meet the criteria for significance. Therefore, we do the Sobel test. The result (0.027) is significantly positive, indicating the mediating effect of industrial structure optimization exists.

Third, the last two columns marked "Information Infrastructure Construction" display the third mechanism. The coefficient *τ*_2_, which represents the influence of information infrastructure construction on economic growth, is positive (0.066) and meets the significant criteria, whereas ORDP’s influence on information infrastructure construction (*β*_1_) is not significant. Hence, we still conduct the Sobel test and the result is 0.060, indicating that ORDP contributes to economic development through information infrastructure construction.

### 5.6 Heterogeneity analysis

Policy effects often diverge due to variations in economic status, resource endowment, human capital, and other factors. Therefore, the conclusions of the baseline regression must be examined for heterogeneity. This section identifies several cities with diverse geographic locations and economic status levels to test the potential heterogeneity consequences of the ORDP on economic growth.

#### 5.6.1 Heterogeneity effects across different regions

In China, local conditions are often emphasized in the process of policy implementation, so policy orientation and support are often heterogeneous in different regions [31]. Due to diverse economic status and resource endowments in different cities, the policy measures vary from one city to another. Assessing ORDP’s heterogeneous effects in different ORDP cities is therefore necessary. Following Yang et al. [4], we classify cities into central and western regions based on their geographic location. Specifically, the Dabie Mountain ORDP cities belong to the central region, while the other two—Left and Right River ORDP cities and Sichuan and Shaanxi ORDP cities—belong to the western region.

Table 8 displays the net policy impact in different regions. We can see that the central region’s ORDP coefficient is significantly positive, with or without the control variables. This result supports our baseline results, which shows that ORDP boosts economic growth. Contrarily, the western region’s ORDP coefficient, albeit positive, is not significant, indicating that ORDP does not influence the western region’s economy. The reason for this insignificant result may be that ORDP cities located in the western region are more difficult to attract capital and talent investment due to their natural conditions, geographic environment, and infrastructure factors [32, 57]. Moreover, lacking adequate financial investment also makes it difficult for them to drive local economic development [44].

**Table 8:** Heterogeneity effects across different regions.

	Central region		Western region	
ORDP	0.057**(0.025)	0.058**(0.025)	0.031(0.028)	0.043(0.027)
Control	No	Yes	No	Yes
City fix effect	Yes	Yes	Yes	Yes
Year fix effect	Yes	Yes	Yes	Yes
R^2^	0.965	0.967	0.961	0.963
Observations	294	294	252	252

*Notes*: The same as Table 2.

#### 5.6.2 Heterogeneity effects across different economic levels

The city’s original economic development characteristics might lead to different policy effects of ORDP on economic performance [58]. Therefore, we use per capita GDP as a criterion to classify cities into high economic level cities and low economic level cities and carry out regression based on Eq (1).

Seeing from Table 9, ORDP’s coefficient in high economic level cities (0.058) is significant at 10%, while the coefficient of ORDP in low economic level cities (0.046) is insignificant, suggesting that implementing ORDP in cities with better economic fundamentals can lead to better economic growth outcomes.

**Table 9:** Heterogeneity effects across different economic levels.

	High Economic Level Cities	Low Economic Level Cities
ORDP	0.058*(0.035)	0.046(0.030)
Control variables	Yes	Yes
City fix effect	Yes	Yes
Year fix effect	Yes	Yes
R^2^	0.965	0.976
Observations	269	263

*Notes*: The same as Table 2.

The following are two possible reasons. For one thing, superior initial economic conditions, such as greater demographic size, larger market scales, and better levels of development enable high-level economy cities to perform better than those low-level economy cities under the same ORDP implementation [51]. For another, low-level economic ORDP cities are mainly located in lagging regions with poor geography [32], making it challenging for ORDP to overpower their initial disadvantageous conditions. Therefore, ORDP can’t significantly boost economic growth in these cities. This is consistent with the studies of Briant et al. [40] and Behaghel et al. [59]. They found that the French Enterprise Zone Program had no impact in physically disadvantaged regions, suggesting that even beneficial policies may not be able to override unfavorable natural characteristics like geography.

## 6. Conclusions

Whether place-based policy can improve economic growth or not has been debated for several decades. In this paper, after selecting 78 prefecture-level cities in the scope of ORDP areas from 2013 to 2019, we study the net impact of ORDP on economic performance using a time-varying DID model. Then, three potential mechanisms and heterogeneous effects behind ORDP on economic growth are further investigated. The findings are as follows: First, ORDP can boost economic growth by 4.01%, but the impact takes a period to emerge and can’t sustain in the long run. The results are reliable after seven robustness tests. Second, expanding government intervention, optimizing industrial structure, and improving information infrastructure construction are three working mechanisms behind ORDP on economic growth. Third, heterogeneity analysis illustrates that ORDP performs better on economic growth in central China cities and high-economy cities.

Hence, we put forward the following three possible recommendations. First, the government needs to maintain the strong push for ORDP implementation. Our analysis of dynamic treatment effects shows that the ORDP’s favorable benefits on economic growth sustained for two years but waned in the fourth year, suggesting that the policy’s effects might eventually wane or even vanish in the long run, similar to other strategies such as the Western Development policy [25]. Learning a lesson from other policies, the government should keep promoting the implementation of ORDP vigorously to maintain the positive effects of the policy. Second, develop a rational industrial development pattern. Our mechanism analysis proves that industrial structure upgrading helps push economic growth in ORBAs. However, currently, the local government in ORBAs tends to rely more on developing the manufacturing industries and thus be trapped in an industry-driven trap, resulting in a shortage of modern service industry. Due to the endowments in ORBAs, especially the abundant Red Tourism Resources, the modern service industry is very suitable for ORBAs and should be further promoted. Third, improve infrastructure construction, especially the information infrastructure. Our mechanism analysis indicates that information infrastructure can effectively improve the positive impact of ORDP on economic development. Therefore, if local governments want to absorb the policy dividend effect in a better way, they need to improve infrastructure construction and supply a better information infrastructure environment such as promoting e-commerce in ORBAs.

## Supporting information

S1 File (XLS)

## References

[1] Candelaria C, Daly M, Hale G. Beyond Kuznets: Persistent regional inequality in China. Federal Reserve Bank of San Francisco Working Paper Series. 2010.

[2] Chen M, Zheng Y. China’s regional disparity and its policy responses. China & World Econ. 2008;16(4): 16–32. doi: 10.1111/j.1749-124X.2008.00119.x

[3] Chen B, Lu M, Timmins C, Xiang K. Spatial misallocation: Evaluating place-based policies using a natural experiment in China. National Bureau of Economic Research. 2019. doi: 10.3386/w26148

[4] Yang Z, Shao S, Xu L, Yang L. Can regional development plans promote economic growth? City-level evidence from China. Socio-Econ Plan Sci. 2021:101212. doi: 10.1016/j.seps.2021.101212

[5] Lu Y, Wang J, Zhu L. Place-based policies, creation, and agglomeration economies: Evidence from China’s Economic Zone Program. American Economic Journal: Economic Policy. 2019;11(3):325–60. doi: 10.1257/pol.20160272

[6] Sun Y, Liao W. Resource-exhausted city transition to continue industrial development. China Econ Rev. 2021;67:101623. doi: 10.1016/j.chieco.2021.101623

[7] Lu Y, Yu L. Trade liberalization and markup dispersion: Evidence from China’s WTO accession. American Economic Journal: Applied Economics. 2015;7(4):221–53. doi: 10.1257/app.20140350

[8] Liu S, Liu C, Yang M. The effects of national environmental information disclosure program on the upgradation of regional industrial structure: Evidence from 286 prefecture-level cities in China. Struct Change Econ D. 2021;58:552–561. doi: 10.1016/j.strueco.2021.07.006

[9] Ren W, Xue B, Yang J, Lu C. Effects of the Northeast China Revitalization Strategy on regional economic growth and social development. Chinese Geogr Sci. 2020;30(5):791–809. doi: 10.1007/s11769-020-1149-5

[10] Wang Z, Wang S, Wang J, Wang Y. Development zones and urban economic performance in China: Direct impact and channel effects. Growth Change. 2022;53(4):1762–1782. doi: /10.1111/grow.12621

[11] Jia J, Ma G, Qin C, Wang L. Place-based policies, state-led industrialisation, and regional development: Evidence from China’s Great Western Development Programme. Eur Econ Rev. 2020;123:103398. doi: 10.1016/j.euroecorev.2020.103398

[12] Liu C, Ma G. Are Place-based policies always a blessing? Evidence from China’s National Poor County Programme. The Journal of Development Studies. 2019;55(7):1603–1615. doi: 10.1080/00220388.2018.1438598

[13] Zhang J, Zhou X. Synthetic control methods for case study: Estimating the economic effect of the Western Development Strategy. The Quantitative Economics Research. 2020;11(01):87–100. doi: 10.16699/b.cnki.jqe.2020.01.007

[14] Yang D, Wang H. The reevaluation of the effect of Northeast China Revitalization Policy: An analysis based on light data and PSM-DID. Business Research. 2021;05:35–44. doi: 10.13902/j.cnki.syyj.2021.05.005

[15] Deng H, Yu Y, Zhao J. Are Place-based policies effective? Evidence from China’s Development Zones. Financial Research. 2019;45(01):4–18. doi: 10.16538/j.cnki.jfe.2019.01.001

[16] Grewal BS, Ahmed AD. Is China’s Western Region Development Strategy on track? An assessment. The Journal of contemporary China. 2011;20(69):161–81. doi: 10.1080/10670564.2011.541626

[17] Rafael LP, Andrei S. Informality and development. The Journal of Economic Perspectives. 2014;28(3):109–126. doi: 10.1257/jep.28.3.109

[18] Pranab B, Dilip M. Capture and governance at local and national levels. Am Econ Rev. 2000;90(2):135–9. doi: 10.1257/aer.90.2.135

[19] Dilip M. Political decentralization. Annu Rev of Econ. 2015;7(1):231–249. doi: 10.1146/annurev-economics-080614-115527

[20] Movshuk O. The reliability of China’s growth figures: A survey of recent statistical controversies. Journal of Econometric Study of Northeast Asia. 2002;35.

[21] Henderson JV, Storeygard A, Weil DN. Measuring economic growth from outer space. Am Econ Rev. 2012;102(2):994–1028. doi: 10.1257/aer.102.2.994 25067841 PMC4108272

[22] Baum-Snow N, Brandt L, Henderson JV, Turner MA, Zhang Q. Roads, railroads, and decentralization of Chinese cities. The Review of Economics and Statistics. 2017;99(3):435–448. doi: 10.1162/REST_a_00660

[23] Elvidge CD, Baugh KE, Kihn EA, Kroehl HW, Davis ER, Davis CW. Relation between satellite observed visible-near infrared emissions, population, economic activity and electric power consumption. Int J Remote Sens. 1997;18(6):1373–1379. doi: 10.1080/014311697218485

[24] Lazar M. Shedding light on the global distribution of economic activity. The open geography journal. 2010;3(1):147–160. doi: 10.2174/1874923201003010147

[25] Jiang F, Wu M, Chen J. Reassessment of the effect of the western development policy—"Quasi-Natural" experimental design based on DMSP/OLS night light data. Investment Research. 2021;40(6):4–22.

[26] Yu B, Tang M, Wu Q, Yang C, Deng S, Shi K, et al. Urban Built-up Area Extraction from Logarithm Transformed NPP-VIIRS Nighttime Light Composite Data. 2018;15(8):1279–83. doi: 10.1109/LGRS.2018.2830797

[27] Criscuolo C, Martin R, Overman HG, Van Reenen J. Some causal effects of an industrial policy. Am Econ Rev. 2019;109(1):48–85. doi: 10.1257/aer.20160034

[28] Becker SO, Egger PH, von Ehrlich M. Effects of EU Regional Policy: 1989–2013. Reg Sci Urban Econ. 2018;69:143–152. doi: 10.1016/j.regsciurbeco.2017.12.001

[29] Kline P, Moretti E. Local economic development, agglomeration economies, and the big push: 100 years of evidence from the Tennessee Valley Authority. Q J of Econ. 2014;129(1):275–331. doi: 10.1093/qje/qjt034

[30] Falck O, Koenen J, Lohse T. Evaluating a place-based innovation policy: Evidence from the innovative Regional Growth Cores Program in East Germany. Reg Sci Urban Econ. 2019;79:103480. doi: 10.1016/j.regsciurbeco.2019.103480

[31] Li B, Yang R, Lu J. Does the Central China’s Rising Strategy lead to policy traps: Empirical evidence based on PSM−DID method. China’s Economic Problems. 2019;3:40–53. doi: 10.19365/j.issn1000-4181.2019.03.04

[32] Han G, Zhao J. Thoughts of poverty alleviation in Old Revolutionary Base Area by Xi Jinping and their guiding significance. Theoretical Journals. 2016;5:4–9. doi: 10.14110/j.cnki.cn-37-1059/d.2016.05.002

[33] Lu H, Liu M, Song W. Place-based policies, government intervention, and regional innovation: Evidence from China’s Resource-Exhausted City program. Resour Policy. 2022;75:102438. doi: 10.1016/j.resourpol.2021.102438

[34] Xing L, Yue G, Jiani H, Jun L. Human capital allocation and enterprise innovation performance: An example of China’s Knowledge-Intensive Service. Research in International Business and Finance. 2021;58:101429. doi: 10.1016/j.ribaf.2021.101429

[35] Sun Z, Zhao L, Wang S, Zhang H, Wang X, Wan Z. Targeted poverty alleviation and households’ livelihood strategy in a relation-based society: Evidence from Northeast China. Int J Env Res Pub He. 2021;18(4): 1747. doi: 10.3390/ijerph18041747 33670155 PMC7916877

[36] State Bardhan P. and development: The need for a reappraisal of the current literature. J Econ Lit. 2016;54(3):862–892. doi: 10.1257/jel.20151239

[37] Leong C, Pan SL, Newell S, Cui L. The emergence of Self-Organizing E-Commerce Ecosystems in remote villages of China. Mis Quart. 2016;40(2):475–484.

[38] Nathaniel HL. Externalities, Information costs, and social Benefit-Cost analysis for economic development: An example from telecommunications. Econ Dev Cult Change. 1984;32(2).

[39] Sun W, Wu J, Zheng S. The consumption-driven effect of place-based industrial policy: An empirical study based on Development Zone Policy. Social Sciences in China. 2020;41(4):44–62. doi: 10.1080/02529203.2020.1844427

[40] Briant A, Lafourcade M, Schmutz B. Can tax breaks beat geography? Lessons from the French Enterprise Zone Experience. American Economic Journal: Economic policy. 2015;7(2):88–124. doi: 10.1257/pol.20120137

[41] WFP E. The role of population in economic growth. SAGE Open. 2017;7(4). doi: 10.1177/2158244017736094

[42] Liu Z. Human capital externalities and rural-urban migration: Evidence from rural China. China Econ Rev. 2008;19(3):521–535. doi: 10.1016/j.chieco.2008.04.001

[43] Fleisher B, Li H, Zhao MQ. Human capital, economic growth, and regional inequality in China. J Dev Econ. 2010;92(2):215–231. doi: 10.1016/j.jdeveco.2009.01.010

[44] Fan Y. The impact of financial development on economic growth in middle-income countries. Journal of International Financial Markets, Institutions and Money. 2019;59:74–89. doi: 10.1016/j.intfin.2018.11.008

[45] Marco P. Financial markets and growth: An overview. Eur Econ Rev. 1993;37:613–622. doi: 10.1016/0014-2921(93)90051-B

[46] Pan D, Hong W, He M. Can campaign-style enforcement facilitate water pollution control? Learning from China’s Environmental Protection Interview. J Environ Manage. 2022;301:113910. doi: 10.1016/j.jenvman.2021.113910 34626950

[47] Beck T, Levine R, Levkov A. Big bad banks? The winners and losers from Bank Deregulation in the United States. The Journal of finance (New York). 2010;65(5):1637–67. doi: 10.1111/j.1540-6261.2010.01589

[48] Louis SJ, Robert JL, Daniel GS. Earnings losses of displaced workers. Am Econ Rev. 1993;83(4):685–709.

[49] Raj C, Adam L, Kory K. Salience and taxation: Theory and evidence. Am Econ Rev. 2009;99(4):1145–1177. doi: 10.1257/aer.99.4.1145

[50] Yang X, Lin S, Li Y, He M. Can high-speed rail reduce environmental pollution? Evidence from China. J Clean Prod. 2019;239:118135. doi: 10.1016/j.jclepro.2019.118135

[51] Gao M, Gu Q, He S. Place-based policies, administrative hierarchy, and city growth: Evidence from China. Econ Model. 2022;115:105952. doi: 10.1016/j.econmod.2022.105952

[52] James JH, Hidehiko I, Petra ET. Matching as an econometric evaluation estimator: Evidence from evaluating a job training programme. Rev Econ Stud. 1997;64(4):605–654. doi: 10.2307/2971733

[53] Pan D, Fan W. Benefits of environmental information disclosure in managing water pollution: Evidence from a quasi-natural experiment in China. Environ Sci Pollut R. 2021;28(12):14764–81. doi: 10.1007/s11356-020-11659-2 33216303

[54] Baron RM, Kenny DA. The moderator-mediator variable distinction in social psychological research: conceptual, strategic, and statistical considerations. J Pers Soc Psychol. 1986;51(6):1173–1182. doi: 10.1037//0022-3514.51.6.1173 3806354

[55] Zheng J, Shao X, Liu W, Kong J, Zuo G. The impact of the pilot program on industrial structure upgrading in low-carbon cities. J Clean Prod. 2021;290:125868. doi: 10.1016/j.jclepro.2021.125868

[56] Yu B. The impact of the internet on industrial green productivity: Evidence from China. Technol Forecast Soc. 2022;177:121527. doi: 10.1016/j.techfore.2022.121527

[57] Zeng L. Research on the effect of revitalization and development policy of the Old Revolutionary Areas—Evidence from Quasi-Natural Experiment. Nanchang: Normal University Jiangxi; 2020.

[58] Alder S, Shao L, Zilibotti F. Economic reforms and industrial policy in a panel of Chinese cities. J Econ Growth. 2016;21(4):305–349. doi: 10.1007/s10887-016-9131-x

[59] Behaghel L, Lorenceau A, Quantin S. Replacing churches and mason lodges? Tax exemptions and rural development. J Public Econ. 2015;125:1–15. doi: 10.1016/j.jpubeco.2015.03.006

